# SANS Studies of the Gallium–Indium Alloy Structure within Regular Nanopores

**DOI:** 10.3390/nano12132245

**Published:** 2022-06-29

**Authors:** Andrei V. Uskov, Elena V. Charnaya, Aleksandr I. Kuklin, Min Kai Lee, Lieh-Jeng Chang, Yurii A. Kumzerov, Aleksandr V. Fokin

**Affiliations:** 1 Physics Department, St. Petersburg State University, 198504 St. Petersburg, Russia; a.uskov@spbu.ru; 2 Joint Institute for Nuclear Research, 141980 Dubna, Russia; kuklin@nf.jinr.ru; 3 Moscow Institute of Physics and Technology, 117303 Moscow, Russia; 4 Instrument Center of Ministry of Science and Technology at National Cheng Kung University, Tainan 70101, Taiwan; mklee@mail.ncku.edu.tw; 5 Department of Physics, National Cheng Kung University, Tainan 70101, Taiwan; ljchang@mail.ncku.edu.tw; 6 Ioffe Institute, 194021 St. Petersburg, Russia; yu.kumzerov@mail.ioffe.ru (Y.A.K.); midbarzin@yandex.ru (A.V.F.)

**Keywords:** SANS, Ga-In eutectic nanoalloy, nanotemplate, segregates

## Abstract

Potential applications of nanolattices often require filling their empty space with eutectic metallic alloys. Due to confinement to nanolattices, the structure of phase segregates in eutectic alloys can differ from that in bulk. These problems are poorly understood now. We have used small angle neutron scattering (SANS) to study the segregation in the Ga-In alloy confined to an opal template with the regular pore network, created by a strict regularity of opal constituents in close similarity with nanolattices. We showed that SANS is a powerful tool to reveal the configuration of segregated phases within nanotemplates. The In-rich segregates were found to have specific structural features as small sizes and ordered arrangement.

## 1. Introduction

Nanolattices consist of nano-size units strictly arranged in regular structures [1,2]. A nanolattice comprises an interconnected network of pores, which have a geometry that follows the order of nano-units packing. For practical applications, the pores can be filled with different substances with properties that are affected by size effects coupled with the surface of constituent elements and by the tortuosity and dimensionality of porous network. It is also expected that an important role might be played by the regularity of pore arrangement; however, this problem is poorly understood. The impact of the complete regularity of confined substance disposition is especially significant for strongly inhomogeneous materials such as eutectic metallic alloys, for instance. Nanolattices have close analogs among more well-known regular porous matrices, such as synthetic silica opals and molecular sieves. Those matrices can be used to understand the specific features of confined eutectic alloys, including the impact of ordered pores arrangement.

Eutectic metallic alloys may be used in numerous applications from soft robotics, wearable electronics, bio-devices, and thermal interfaces to self-healing superconductors [3,4,5,6,7,8]. Among these, gallium-based alloys are considered the most suitable nanomaterials due to their stable electrical properties and non-toxicity. Binary Ga-In and ternary Ga-In-Sn nanoalloys demonstrated an especially great potential.

A nanostructured liquid Ga-In alloy has some specific features, which distinguish it from its bulk counterpart: it undergoes a liquid–liquid transition upon supercooling [9], its atomic mobility slows down [10], and its solidus and liquidus lines shift to lower temperatures [11]. When crystallized, the Ga-In nanoalloy segregates into two phases, an In-rich solid solution and a phase consisting of gallium with a very low amount of dissolved indium, in agreement with the phase diagram of the bulk gallium–indium alloy [12]. The shapes and sizes of the segregated phases in the frozen Ga-In alloy confined to nanoporous templates have never been studied because of experimental difficulties; therefore, the relation between pore geometry and distribution of the segregates has not been discussed.

Here, in the particular case of Ga-In, we show that the configuration of the segregates in a frozen eutectic alloy within a nanoporous template can be ascertained using small-angle neutron scattering (SANS). For this, we used loaded samples with two close Ga-In compositions and studied the difference in neutron scattering between them. As far as we know, the approach to the treatment of measurements of a difference in small-angle neutron scattering by two highly heterogeneous solid samples has not been used before. However, this approach has quite similar background ideas to SANS contrast matching. This technique is well-known, and it was applied to an analysis of structure in biologic objects and chemical processes in porous MCM-41 using the Guinier approximation [13,14]. In the present work, we combined this approach with Monte Carlo modeling objects with complex architecture that were also used in [15,16].

## 2. Materials and Methods

**Figure 1:**
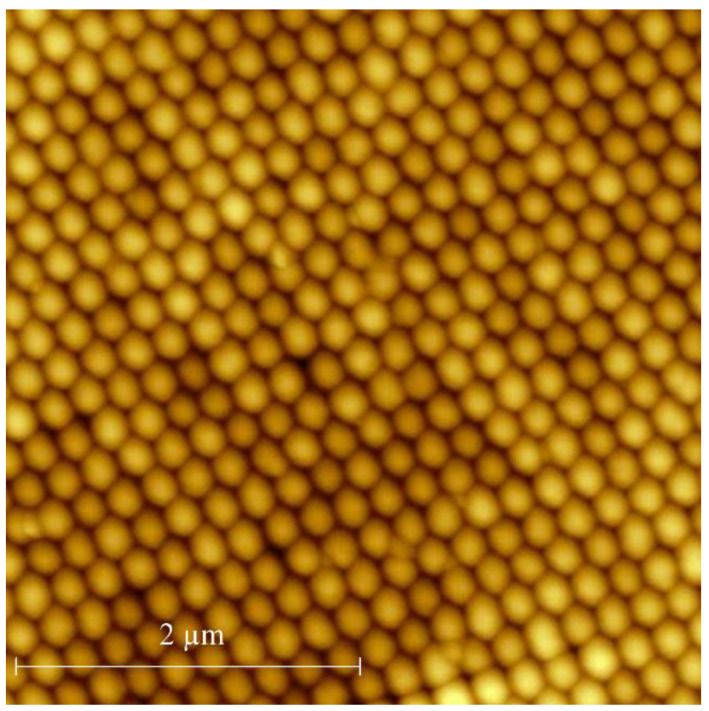
AFM image of the opal surface.

The synthetic opal matrix used in the present paper is a close-packing of silica balls with a mean diameter of 220 nm according to atomic force microscopy (AFM). The AFM image is shown in Figure 1.

The ideal close-packing of rigid spheres has two kinds of pores: octahedral and tetrahedral. The total pore volume is 26%. The pores form a thoroughly interconnected 3 d network. The sizes of the pores, defined as diameters of inscribed spheres, are equal to 0.414 *D* and 0.225 *D* for the octahedral and tetrahedral pores, respectively, where *D* is the diameter of the silica balls. Thus, the pore sizes are about 91 and 49 nm for the opal used. Sintering might slightly reduce the pore sizes. The two different alloy compositions were 94 at.% Ga/6 at.% In and 96 at.% Ga/4 at.% In. The alloys were introduced into the opal pores at a temperature above the liquidus lines under a pressure of up to 10 kbar and then cooled down to room temperature. The plates (about 2 mm-thick) for SANS studies were cut from the loaded opals and cleaned from bulk alloys. Samples with the indium compositions 4 and 6 at.% are referred to hereinafter as S-In4 and S-In6, respectively. While the opal templates, which pores are filled with the alloys, practically do not adsorb water, the samples were vacuumed before the SANS experiments to prevent any impact of water on scattering intensity. Note that strong neutron scattering by water because of a large scattering length density contrast with amorphous silica did not allow for the analysis of the nanostructured Ga-In alloy based on the comparison between empty and loaded templates.

SANS experiments were carried out at the IBR-2 pulsed reactor, Frank Laboratory of Neutron Physics JINR, Dubna. The experimental setup used time-of-flight (TOF) method to determine wavelength. The wavelength accuracy was about 1%. The reactor has PuO_2_ in the active zone and works in pulse mode due to rotating neutron reflector. A chopper was used to form beams with average wavelength 1.2 Å and FWHM 0.7 Å. The wavelength corresponding to every registered count was determined by time delay. The obtained data were programmatically processed. Two detectors located at 12 and 17 m were used to obtain the scattering vector *Q* range from 0.007 to 0.5 Å^−1^. A neutron beam was collimated with cadmium plates to a diameter of 14 mm. The exposure time was 30 min.

The SANS experiments were performed at a temperature of 278 K below the solidus in the confined Ga-In alloy [11]. To avoid disturbances caused by supercooling, the samples were first cooled down to 200 K and then warmed up to the measurement temperature.

## 3. Results

**Figure 2:**
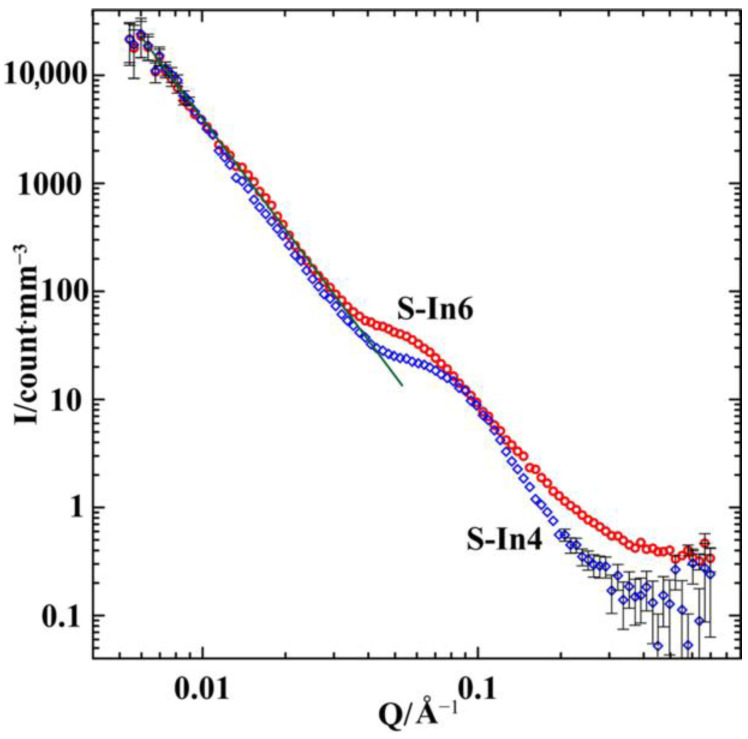
Normalized SANS intensities in the samples with alloy compositions 96 at.% Ga/4 at.% In (S-In4, blue diamonds) and 94 at.% Ga/6 at.% In (S-In6, red circles). The straight green line corresponds to the *I*(*Q*)~*Q*^−3.2^ dependence.

The neutron scattering curves obtained for S-In4 and S-In-6 are shown in Figure 2 in the double-logarithmic scale. These curves were obtained by subtracting the scattering from the empty furnace from the experimental scattering data.

In the whole *Q* range, the scattering intensity in the S-In6 sample with a higher indium amount prevails over that in S-In4. This agrees with scattering lengths listed in [17]. At *Q* < 0.037 Å^−1^, the scattering intensity versus the scattering vector follows the power law *I*(*Q*)~*Q*^−3.2^ for both samples. This is an indication of the fractal character of the samples with fractal dimension *Dm* = 3.2 on a scale larger than 17 nm [18]. The fractality of opal matrices was discussed in [19]. At *Q* > 0.3 Å^−1^ the scattering intensity is comparable with background and incoherent neutron scattering. The scattering curves in Figure 2 demonstrate broad interference maxima in the intermediate ranges of *Q*. The peak in intensity is centered at about 0.07 Å^−1^ for S-In4 and 0.055 Å^−1^ for S-In6. The peak positions provide approximate estimates for the distance d between regular structural heterogeneities according to the Bragg’s law d=2π/Q: d≈ 9 nm and 12 nm for S-In4 and S-In6, respectively. Such an approach, combined with the Guinier law, was used in [20] to treat the scattering by opal matrices loaded with different materials. Since the scattering intensity depends on the distance between scatterers and their sizes and shapes, the above estimates do not take into account the real variations in the form factor with Q. A more accurate analysis requires scattering by an empty opal and correlations between the opal matrix and segregates in the alloy. Due to the impact of the form factor, a shift between peak positions for S-In4 and S-In6 can be related to changes in the arrangements or/and forms of scatterers. Nevertheless, we note that the above estimates for d in S-In4 correlate with the results obtained in the present work.

**Figure 3:**
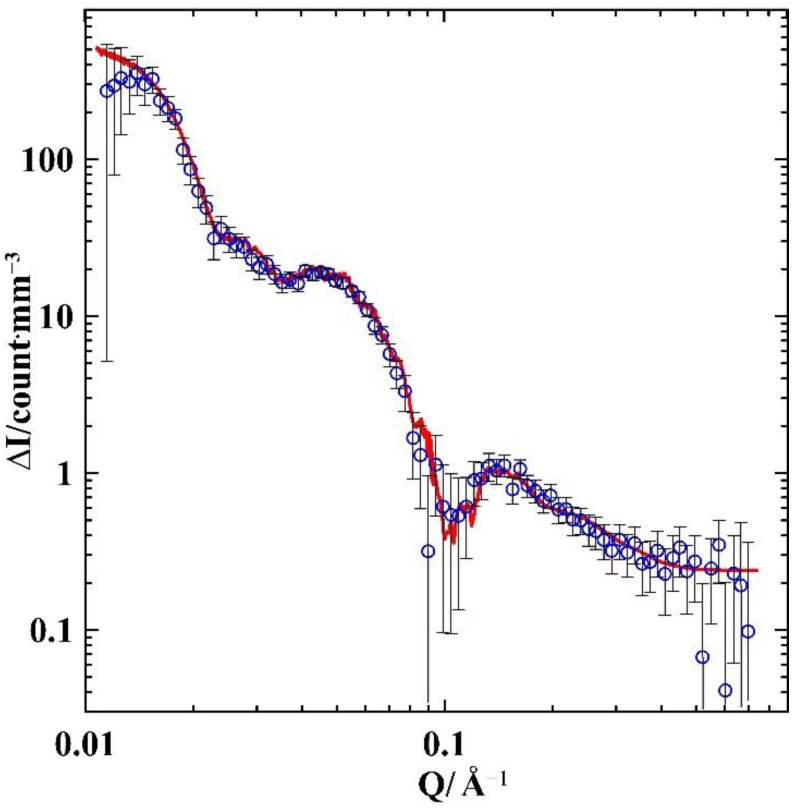
The dependence of $\Delta I\left(Q\right)$ on Q. Blue symbols are data calculated from experiments. Red line is the theoretical fit.

The difference between the two scattering curves is shown in Figure 3. This is wholly due to the different alloy composition in the samples, which lead to different configuration in the segregates.

## 4. Discussion

To treat the scattering by the loaded opal matrices on the base of data presented in Figure 2 we need the contribution of the empty opal. However, the empty opal matrices comprise an amount of adsorbed water up to 5% of the pore volume, which affects the scattering intensity. This poses problems for correcting the evaluation of the opal matrix contribution to the total scattering intensity. Another way consists of filling the opal pores with water, measuring the filled opal matrix and renormalizing the obtained SANS data taking into account the contrast between silica and water. The drawbacks of this method are related to closed voids between the second order spheres in the opal structure [19], which are not filled with water. On the contrary, knowledge of the scattering by the empty opal is not required for an analysis of the difference between the scattering intensities of two samples with alloys of similar compositions. 

Now we consider a model which is used to treat the data presented in Figure 3. The scattering intensity $I(\vec{Q})$ of a sample consisting of a porous opal template with solid eutectic alloy is given by:

(1) $$I\left(\vec{Q}\right)={\left|\int_{V_{M}}\rho_{M}\left(\vec{r}\right)e^{i\vec{Q}\vec{r}}d\vec{r}+\int_{V_{I}}\rho_{I}e^{i\vec{Q}\vec{r}}d\vec{r}+\int_{V_{II}}\rho_{II}e^{i\vec{Q}\vec{r}}d\vec{r}\right|}^{2},$$

where $V_{M}$, $V_{I}$, and $V_{II}$ are volumes of silica balls and of segregated phases enriched with Ga and In, respectively; $\rho_{M}\left(\vec{r}\right)$, $\rho_{I}$, $\rho_{II}$ are relevant scattering length densities. It should be emphasized that Equation (1) cannot be simplified using a model of solitary scatterers, which is justified when the distance between the scatterers is much larger than the scatterer size. In our case, the distances between the inhomogeneities in the samples are comparable with their sizes.

Based on Equation (1), we can obtain the difference $\Delta I\left(\vec{Q}\right)$ in the scattering intensities by the samples with different alloy compositions:

(2) $$\Delta I\left(\vec{Q}\right)=I_{S-In6}(\vec{Q})-I_{S-In4}(\vec{Q})$$

To facilitate the analysis of $\Delta I\left(\vec{Q}\right)$, we transformed Equation (2) using the Babinet’s principle, according to which the diffraction intensity does not change when scattering density lengths of all sample constituents change by a constant [21]. Following the Babinet’s principle, we decreased the scattering density lengths of the opal matrix and segregates by: $\rho_{I}$:${\rho^{\prime }}_{I}=0$, ${\rho^{\prime }}_{II}=\rho_{II}-\rho_{I}$, ${\rho^{\prime }}_{M}(\vec{r})=\rho_{M}(\vec{r})-\rho_{I}$.

The total amount of phase II elements in S-In6 is higher than in S-In4 because of a larger indium concentration. Let us denote the additional volume occupied by the phase II elements in S-In6 by ΔV, keeping $V_{II}$ for the volume of the phase II in S-In4, and transform Equation (2) to:

(3) ΔIQ→=∫ΔVρ′IIeiQ→r→dr→2+2Re∫VMρ′MeiQ→r→dr→⋅∫ΔVρ′IIeiQ→r→dr→¯+∫ΔVρ′IIeiQ→r→dr→⋅∫VIIρ′IIeiQ→r→dr→¯

Here, we suggest that the increase in the indium composition is not associated with the decrease in element size. This suggestion is based on Figure 2. Since the intensity peak for S-In6 moves to a lower Q compared to that in S-In4, it implies a ~30% increase in the sizes of the phase II elements (which affect the form factor) or a ~30% increase in the distance between the elements (which affects the S-factor). The latter scenario, together with a noticeable decrease in sizes of the phase II elements, means that the elements cannot be held within pores. Note that we do not consider a scenario when both the distance between elements and their sizes increase as it requires too many fitting parameters.

The first term in Equation (3) does not depend on the mutual disposition of the matrix constituents, phase II elements emerged in S-In4, and additional phase II elements emerged in S-In6. The second term takes into account the mutual disposition of segregates in both samples and the matrix. This term can be simplified if we suggest that the volume ΔV consists of a large number of separated elements with arbitrary sizes and shapes. In this case, averaging over the sample volume yields the first item in the parentheses, which is equal to zero when the sample size is much larger than 1/Q. Then, Equation (3) can be written:

(4) ΔIQ→=∫ΔVρ′IIeiQ→r→dr→2+2Re∫ΔVρ′IIeiQ→r→dr→⋅∫VIIρ′IIeiQ→r→dr→¯

Equation (4) shows that the change in the scattering intensity upon the increasing fraction of indium in S-In6 depends on the mutual arrangement of phase II segregates of various sizes and shapes. Denote the volume of an phase II element in S-In4 $\Delta U_{j,l}$ and the volume of an additional element in S-In6 $\Delta V_{i,k}$, where the first index indicates the type (shape and size) of the element and the second index indicates its number among similar elements. The integrals in Equation (4) can be transformed:

(5) $$\int_{\Delta V}{\rho^{\prime }}_{II}e^{i\vec{Q}\vec{r}}d\vec{r}=\sum_{i}\int_{\Delta V_{i,0}}{\rho^{\prime }}_{II}e^{i\vec{Q}\vec{r}}d\vec{r}\sum_{k}e^{i\vec{Q}\left({\vec{R}}_{i,k}-{\vec{R}}_{i,0}\right)}$$

(6) $$\int_{V_{II}}{\rho^{\prime }}_{II}e^{i\vec{Q}\vec{r}}d\vec{r}=\sum_{j}\int_{\Delta U_{j,0}}{\rho^{\prime }}_{II}e^{i\vec{Q}\vec{r}}d\vec{r}\sum_{l}e^{i\vec{Q}\left({\vec{R}}_{j,l}-{\vec{R}}_{j,0}\right)}$$

assuming the identity of the elements with similar shapes and sizes. Here, ${\vec{R}}_{i,k}$ and ${\vec{R}}_{j,l}$ are the position vectors of the elements. Then, Equation (4) is given:

(7) ΔIQ→=∑i∫ΔVi,0ρ′IIeiQ→r→dr→∑keiQ→R→i,k−R→i,02+2Re∑i∫ΔVi,0ρ′IIeiQ→r→dr→∑keiQ→R→i,k−R→i,0⋅∑j∫ΔUj,0ρ′IIeiQ→r→dr→∑leiQ→R→j,l−R→j,0¯

We used the obtained Equation (7) to fit the curve for the scattering intensity difference shown in Figure 3. Let us first consider the crystallization process of the Ga-In alloy within pores. According to the phase diagram [12], the composition of the alloy studied in the present work is shifted from the eutectic point, which corresponds to 14.2 at.% In in bulk. Provided that the eutectic point did not remarkably change for the alloy within opal pores, the excess of gallium starts precipitating at the liquidus temperature. As the crystallization of liquids under nanoconfinement is induced by the inner pore surface, the gallium-rich solid phase segregates near the pore walls. The fraction of this phase grows with the decreasing temperature. Just above solidus, we have the gallium-rich solid phase I and liquid alloy with eutectic composition, surrounded by phase I. The melt of eutectic composition comprises 28% of the total number of atoms in S-In4 and 42% in S-In6. Below solidus, the liquid fraction segregates with emergence of the phase I and indium-rich phase II. Then, crystalline elements of phase II are separated from the pore walls. In the bulk alloy, the phase II segregates as lamellae or rods. In agreement with these types of segregates, we assumed that elements of phase II in S-In4 were shaped as spheres or ellipsoids (as approximations of lamellae) and cylinders (as approximations of rods). We also considered various arrangements of elements: randomly located elements, elements in nodes of cubic or hexagonal close packings.

Upon increasing the concentration of indium in S-In6, new elements can appear or indium shells can grow around the elements existing in S-In4. For each element of a particular shape, we analytically calculated the integrals $\int_{\Delta V_{i,0}}{\rho^{\prime }}_{II}e^{i\vec{Q}\vec{r}}d\vec{r}$ and $\int_{\Delta U_{j,0}}{\rho^{\prime }}_{II}e^{i\vec{Q}\vec{r}}d\vec{r}$. Then, we took the position vectors of the elements by the Monte Carlo method and numerically calculated Equation (7). The amount of elements corresponds to the alloy compositions. This procedure was repeated until a perfect agreement between the experimental and computed data was achieved. Figure 3 shows a peak in the experimental $\Delta I\left(\vec{Q}\right)$ dependence near Q~0.06 Å^−1^. It indicates a typical length in the samples of approximately 13 nm. This length may correspond to a number of phase II elements with such a size or to a distance between smaller elements in the case of their regular disposition. We used this length as an initial estimate in our calculations for various versions of the shape and arrangement of elements.

The following general assumptions were used in our calculations. The phase II elements in S-In4 can be spheres and/or spheroids. They do not touch with the pore surface. Their arrangement can be random, ordered, or partially ordered. When the ordered or partially ordered arrangement of elements is considered, this arrangement can correlate or not with the disposition of the opal spheres. The phase II volume in S-In6 can increase due to the increase in the number of elements or/and increase in their radii. The calculations were carried out for the volume 10 × 10 × 10 µm. Every iteration was repeated 1000 times with random orientations of the opal template to account for the real opal mosaic structure.

**Figure 4:**
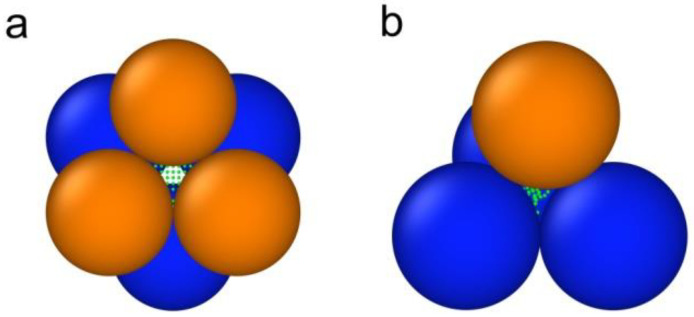
The cartoon view of spherical phase II elements within octahedral (**a**) and tetrahedral (**b**) pores.

The best result is shown in Figure 3. The corresponding model is as follows. Most of the phase II segregates are spherical elements. In S-In4 the mean radius of spheres is 3.1 nm. The radii obey the Gaussian distribution with a standard deviation 0.4 nm. When the indium concentration increased, the radii of spherical elements also increased. The increases in the radii in S-In6 were assumed to be proportional to the initial radii in S-In4. The factor of proportionality is 1.147. The spherical elements in both samples were set in the nodes of the cubic close-packing. The mean distance between the nearest elements confined to every pore was 13.7 nm with a standard deviation of 3.2 nm. Note that the distance obtained was rather close to the estimate on the base of the Bragg maximum position. As the sizes of the elements are comparable with the distance between the elements, the influence of the form factor was noticeable. This explains the difference between the estimate and the calculated distance. Axes of the close packing structures were randomly oriented. The positions of spherical elements can shift relative to the nodes in the close-packing. The shifts also follow the normal distribution with a standard deviation of 2 nm. The spherical elements in the octahedral and tetrahedral pores are shown in Figure 4. A small amount of the phase II forms larger spheroid elements, which major and minor axes in S-In4 are 46 and 36 nm, respectively. The major axis rises up to 53 nm in S-In6, and the sizes of spheroids are fixed in each sample. The total volume of spheroids is 2.4% of the total volume of phase II in both samples. These larger elements segregate in the octahedral pores. Their positions are random. The parameters used in the model, which provides the fit in Figure 3, are listed in Table 1.

Figure 3 shows a perfect agreement between the experimental results by SANS and fitting. All other variants of shapes, sizes, and distribution yield poor fits. It should be noted that the central point of the fitting model is a quite a regular arrangement of the phase II spherical elements, which causes the emergence of a diffused peak near Q~0.06 Å^−1^. In contrast, our preliminary studies of irregular porous templates loaded with Ga-In alloys of the same compositions did not reveal any regularity in the arrangement of segregates.

## 5. Conclusions

We showed for the first time that small-angle neutron scattering (SANS) can be used for studies of the segregate structure in eutectic metallic alloys embedded in nanoporous templates. Such findings are important for applications of various nanolattices loaded with metallic alloys, e.g., gallium indium alloy. The suggested method requires samples with two close alloy compositions.

An analysis of the difference in SANS intensity for the samples with the Ga-In alloys containing 4 and 6 at.% In revealed the emergence of regularly packed nanosegregates within opal pores in striking contrast to the bulk counterparts with segregates on the micron scale.

## Figures and Tables

**Table 1:** Parameters of the model, corresponding to the fit in Figure 3.

Sample	S-In4	S-In6
Shape of elements	sphere	sphere
Total fraction of phase II	97.6%	97.6%
Mean radius	3.1 nm	3.6 nm
Standard deviation of radius	0.4 nm	0.5 nm
Distance between nearest elements	13.7 nm	13.7 nm
Standard deviation of distance	3.2 nm	3.2 nm
Shape of elements	Spheroid	Spheroid
Total fraction of phase II	2.4%	2.4%
Major axis	46 nm	53 nm
Minor axis	36 nm	36 nm

## Data Availability

Not applicable.
